# Ultrasensitive Detection of RNA and DNA Viruses Simultaneously Using Duplex UNDP-PCR Assay

**DOI:** 10.1371/journal.pone.0141545

**Published:** 2015-11-06

**Authors:** Yong Huang, Na Xing, Zengguo Wang, Xiujuan Zhang, Xiaomin Zhao, Qian Du, Lingling Chang, Dewen Tong

**Affiliations:** College of Veterinary Medicine, Northwest A&F University, Yangling, Shaanxi, 712100, P. R. China; University of Florida, UNITED STATES

## Abstract

Mixed infection of multiple viruses is common in modern intensive pig rearing. However, there are no methods available to detect DNA and RNA viruses in the same reaction system in preclinical level. In this study, we aimed to develop a duplex ultrasensitive nanoparticle DNA probe-based PCR assay (duplex UNDP-PCR) that was able to simultaneously detect DNA and RNA viruses in the same reaction system. PCV2 and TGEV are selected as representatives of the two different types of viruses. PCV2 DNA and TGEV RNA were simultaneously released from the serum sample by boiling with lysis buffer, then magnetic beads and gold nanoparticles coated with single and/or duplex specific probes for TGEV and PCV2 were added to form a sandwich-like complex with nucleic acids released from viruses. After magnetic separation, DNA barcodes specific for PCV2 and TGEV were eluted using DTT and characterized by specific PCR assay for specific DNA barcodes subsequently. The duplex UNDP-PCR showed similar sensitivity as that of single UNDP-PCR and was able to detect 20 copies each of PCV2 and TGEV in the serum, showing approximately 250-fold more sensitivity than conventional duplex PCR/RT-PCR assays. No cross-reaction was observed with other viruses. The positive detection rate of single MMPs- and duplex MMPs-based duplex UNDP-PCR was identical, with 29.6% for PCV2, 9.3% for TGEV and 3.7% for PCV2 and TGEV mixed infection. This duplex UNDP-PCR assay could detect TGEV (RNA virus) and PCV2 (DNA virus) from large-scale serum samples simultaneously without the need for DNA/RNA extraction, purification and reverse transcription of RNA, and showed a significantly increased positive detection rate for PCV2 (29%) and TGEV (11.7%) preclinical infection than conventional duplex PCR/RT-PCR. Therefore, the established duplex UNDP-PCR is a rapid and economical detection method, exhibiting high sensitivity, specificity and reproducibility.

## Introduction

Along with the development of large-scale and intensive swine production, mixed infections of multiple pathogens are increasingly becoming common in swine farms. Porcine reproductive and respiratory syndrome virus (PRRSV), porcine circovirus type 2 (PCV2), classical swine fever virus (CSFV), porcine pseudorabies virus (PRV), transmissible gastroenteritis virus (TGEV), porcine parvovirus (PPV) and porcine epidemic diarrhea virus (PEDV) are major pathogens causing heavy economic losses in swine industry [1–8]. On the basis of clinical signs, it is difficult to determine whether sick pigs are infected by single or multiple viruses [9]. Therefore, it is imperative to establish an effective and rapid method to detect multiple DNA and RNA viruses simultaneously in single sample.

Traditional diagnostic methods of DNA and RNA viruses are mainly dependent on detection of viral proteins and nucleic acids. Currently, common methods for detecting viral antigen in solution is enzyme-linked immunosorbent assay (ELISA), but ELISA shows some shortcomings that are difficult to overcome. For example, ELISA requires at least one highly specific antibody for a particular viral antigen, but a specific antibody is not able to detect all viral strains of a kind of virus due to genotype difference; a ELISA kit is usually for one kind of virus, leading to that ELISA detection are complicated, time-consuming and expensive, and it is difficult to achieve scale detection when we need to detect a variety of viruses at the same time. In addition, it is difficult to find viral infection by ELISA when viral load is below a certain level because the sensitivity base line of ELISA is relatively higher [10, 11]. The nucleic acids-based detection methods include conventional PCR, RT-PCR, loop-mediated isothermal amplification (LAMP) and real-time PCR, which have been used in the diagnosis of virus infection [12–16]. Although LAMP and real-time PCR are more sensitive than conventional PCR or RT-PCR, LAMP can only detect one pathogen at a time and LAMP products are difficult to identify, while real-time PCR requires special or expensive instruments and easily shows false positive results [17, 18]. However, more critical is that all the existing PCR-based assays need RNA/DNA extraction. It is known that the extraction and detection procedures of DNA and RNA are different from each other. RNA is easily degradable as compared to DNA, so in PCR-based methods of detecting RNA viruses, viral genomic RNA extracted from field samples should be utilized to synthesize complementary DNA (cDNA) first, which are time-consuming and labor-intensive.

UNDP-PCR is an ultrasensitive nanoparticle DNA probe-based PCR method, in which magnetic microparticles (MMPs) coated with virus specific DNA probes and gold nanoparticles (AuNPs) coated with virus specific oligonucleotides are used to enrich virus genomes from samples and form an AuNP-RNA/DNA-MMP complex. Then the specific oligonucleotides are released and characterized by PCR after the complex is washed. In the previous study, we established this method to detect DNA virus PCV2, which exhibited a detection limit of 2 copies of PCV2 genomic DNA and 10 copies of PCV2 in serum that is 500-fold more sensitive than conventional PCR [19]. However, it is still needed to test whether this method can be used in the detection of RNA virus. In this study, we aimed to develop a method for simultaneous detection of preclinical DNA and RNA virus mixed infection in the same reaction system based on UNDP-PCR method. TGEV and PCV2, as the representatives of RNA and DNA viruses respectively chosen from a variety of viruses related to porcine diseases, were used to establish duplex UNDP-PCR assays. PCV2, a DNA virus with a circular genome of 1.7 kb, has been reported to cause wide infection throughout the world and serious damage to pig producers, while TGEV, an enveloped virus with a positive-stranded RNA genome, has been recognized as a principal causative agent of enteric disease [20, 21]. Firstly, the UNDP-PCR assay for TGEV was developed and identified. Then, single MMPs-based or duplex MMPs-based duplex UNDP-PCR assays for both PCV2 and TGEV was developed. MMPs coated with specific DNA probes for either TGEV or PCV2 (single MMPs), or for both TGEV and PCV2 (duplex MMPs) were used to enrich TGEV and PCV2 viral genomes from serum samples, and AuNPs coated with optimal oligonucleotides (oligo) specific for either TGEV or PCV2 were used to magnify weak signals from very low level of TGEV/PCV2 virus enriched by MMPs from serum samples. The duplex UNDP-PCR assay is suitable for simultaneous detection of RNA and DNA viruses in early viral infection, providing an effective approach for diagnosis of swine diseases.

## Materials and Methods

### Viruses and cells

TGEV strain (GenBank No. HQ462571), PCV2 strain (GenBank No. EU366323), PPV YL strain (GenBank No. JN860197), PEDV strain (GenBank No. AF353511) and PRRSV Shaanxi strain (GenBank No. HQ401282) used in this study were isolated and purified previously by our team and stocked in our laboratory [22–26]. The CSFV Shimen Strain (GenBank No.AY775178) was provided kindly by Professor Yanming Zhang [27]. These virus strains were maintained at -80°C and used as standard viruses for this study. TGEV, PCV2 and PPV were propagated in PK-15 cells. CSFV was propagated in ST cells. PEDV was propagated in Vero cells. PRRSV was propagated in Marc-145 cells. Four types of cells (PK-15, ST, Vero, and Marc-145) were maintained in Dulbecco’s modified eagle medium (DMEM) (Gibco, Gaithersburg, MD, USA) supplemented with 10% heat inactivated fetal calf serum (Gibco).

### Field samples

During the period from Nov, 2014 to Jun, 2015, 162 serum samples were collected from healthy pigs in pig-producing farms near Xianyang and Baoji of Shannxi province, China. The pigs were humanely euthanized by injecting with 15 mg/kg of Ketamine in the jugular vein, then 3–5 ml of blood samples were collected from each pig by jugular venipuncture. The serum samples were tested using the conventional PCR/RT-PCR assay and single or duplex UNDP-PCR. The experiment was approved by the Ethical Committee for Animal Experiments of the Northwest A&F University and performed according to the Animal Ethics Procedures and Guidelines of the People’s Republic of China. No other specific permissions were required for this study.

### Purification of viral DNA or RNA

Viral RNA/DNA Kit (OMEGA, USA) was used to extract and purify viral genomic DNA or RNA from virus-infected cell cultures or serum samples according to the manufacture’s protocol. Then the extracted viral RNA was reverse transcribed into the complementary DNA (cDNA) using the reverse transcriptase kit (Takara Corp., Japan) according to manufacturer’s instructions.

### Primers and probes

In this study, specific TGEV DNA probes and oligonucleotides used in UNDP-PCR were designed via multiple sequence alignment of complete genomes of ORF1a from various TGEV strains published on National Center for Biotechnology Information using VECTOR NTI 9 and DNASTAR software package. Primers for conventional RT-PCR method of detecting TGEV and PEDV were designed using primer-blast software on the basis of TGEV and PEDV highly conserved region of ORF1a. Primers for conventional PCR/RT-PCR detection of PCV2, PPV, PRRSV and CSFV were designed as described in previous studies [14, 19]. All the primers, probes and oligonucleotides designed for this experiment owned higher specificity to make sure diagnosis more precise. The primers, probes and oligonucleotides presented in Table 1 were synthesized by Sangon (Shanghai, China).

**Table 1:** Primers and probes used in this study.

Label	Orientation	Assay	Sequence(5’-3’)	Position
PEGFP-F1	Forward	LINK-PCR	ATAGCGGTTTGACTCACGGG	400–419
PEGFP-R1	Reverse	LINK-PCR	CCCTTTGACGTTGGAGTCCA	1839–1820
PEGFP-F2	Forward	LINK-PCR	CTGGGGTTCGAAATGACCGA	3432–3451
PEGFP-R2	Reverse	LINK-PCR	GCAGCCACTGGTAACAGGAT	4270–4251
PEDVF	Forward	Conventional-PCR	GTGGTAACATCGTGCCAGT	622–640
PEDVR	Reverse	Conventional-PCR	AGTTGACCGTCTTCGGAGT	736–718
TGEVF	Forward	Conventional-PCR	CGTAATGGTGACAGCCGAGT	4554–4573
TGEVR	Reverse	Conventional-PCR	AGCAGCATCACGGAAACCAT	4988–4969
PCV2-DP1	Forward	Detect-PCR	AGCAGAAGAACGGCATCAAG	
PCV2-DP2	Reverse	Detect-PCR	GCCATCTTGGCCAGATCCT	
TGEV-DP1	Forward	Detect-PCR	AGATTTCGATTCCACCGCCG	
TGEV-DP2	Reverse	Detect-PCR	GAAGACTAGGCACGTTGAC	
Probe 1		Hybridization	5’NH_2_-T_15_CAAATATTTCGTTTAGTTCGAGTTGGTGTCCG	78–109
Probe 2		Hybridization	5’NH_2_-T_15_CGAATAGGAAGACTAGGCACGTTGAC	360–385
Probe 3		Hybridization	5’NH_2_-T_15_ACTCATCATAAGAAGCCAAACAGGCTTTGCAT	6146–6177
Probe 4		Hybridization	5’NH_2_-T_15_GTGTCTGCCTTGCAGTCCTAGAGCAC	6494–6519
Probe 5		Hybridization	5’NH_2_-T_15_TTAGACAGTTATTAACTGGTTGCATGCCTCTC	11999–12026
Probe 6		Hybridization	5’NH_2_-T_15_CGAATTGGATCTTGTGTGCCAGTTGG	12201–12226
Oligo 1		Hybridization/Amplification	5’SH-T_15_CAAATATTTCGTTTAGTTCGAGTTGGTGTCCGTCATGAGATTATCAAA	
Oligo 2		Hybridization/Amplification	5’SH-T_15_CGAATAGGAAGACTAGGCACGTTGACTCATGAGATTATCAAA	
Oligo 3		Hybridization/Amplification	5’SH-T_15_ACTCATCATAAGAAGCCAAACAGGCTTTGCATTCATGAGATTATCAAA	
Oligo 4		Hybridization/Amplification	5’SH-T_15_GTGTCTGCCTTGCAGTCCTAGAGCACTCATGAGATTATCAAA	
Oligo 5		Hybridization/Amplification	5’SH-T_15_TTAGACAGTTATTAACTGGTTGCATGCCTCTCTCATGAGATTATCAAA	
Oligo 6		Hybridization/Amplification	5’SH-T_15_CGAATTGGATCTTGTGTGCCAGTTGGTCATGAGATTATCAAA	

### Preparation of MMPs coated with TGEV and / or PCV2 specific probes for UNDP-PCR assay

In the presence of water-soluble N-EthylN9-[3-dimethylaminopropyl] carbodiimide hydrochloride (EDC), TGEV and/ or PCV2 specific oligonucleotide probes modified with 5’ amino (NH2) were bond to the carboxylated-modified MyOne Dynabeads to form a peptide bond. The detailed steps of the assay were according to manufacturer’s instruction [28]. Then the functionalized MMPs were resuspended in Tris-EDTA (TE) buffer to a concentration of 10 mg/ml and stored at 4°C until required.

### Preparation of AuNPs coated with TGEV or PCV2 specific oligos

AuNPs coated with TGEV or PCV2 specific oligo were prepared as described in previous study [19], briefly, 1 ml of 15 nm-diameter AuNPs (10 nmol/L) were washed and resuspended in 100 μl sterile deionized water. Then, the 5’ sulfydryl (SH)-modified TGEV/PCV2 specific oligonucleotides were added and mixed with AuNPs to establish covalent Au-S bond. In the binding process of thiolated AuNPs and SH-modified oligonucleotides, a final concentration of 0.01 M PBS (0.1 M NaCl in 0.01 M of phosphate buffer, PH 7.0) was developed by adding 0.1 M PBS to the reaction tube three times to incubate for more than 48 hours at room temperature. Then, unbound DNA probes were removed by washing twice with 0.01 M PBS. Finally, the prepared functionalized AuNPs were stored in 0.01 M PBS at 4°C until used.

### Hybridization reaction and PCR detection of UNDP-PCR

Viral serum samples were mixed with same volume of lysis buffer (0.01 M Tris-HCl, 1 mM EDTA, 0.015 M NaCl, 0.5% SDS, pH 8.0), and then boiled for 15 minutes to release viral RNA or DNA. The products were mixed with 2 μl of probe-coated MMPs and 5× hybridization buffer (5×SSC, 0.1% Tween-20 and 2% SDS in H_2_O). The mixture of these components was incubated for 30 minutes by stirring. Subsequently, 2 μl of functionalized AuNPs were added, followed by incubation at 50°C for 40 minutes. The sandwich-like AuNP-RNA/DNA-MMP complexes in the tube were separated using magnetic wells and washed twice with 1 ml of hybridization buffer and twice with 1 ml TE buffer to remove remaining hybridization buffer and unbound probes-functionalized MMPs and oligos-functionalized AuNPs.

The oligonucleotides on the surface of gold nanoparticles were eluted using 100 μl of elution buffer (0.5 M DTT, 10 mM Tris-HCl, 1 mM EDTA, pH 7.5) for 10 minutes at room temperature. Then, the eluted oligonucleotides were precipitated with NaAc and absolute alcohol. The precipitated oligonucleotides were mixed with specific capture ssDNA and detected by PCR using specific detect-PCR primers for PCV2 and/or TGEV in Table 1. The PCR assay was performed as described in the previous study [19]. The PCR products were separated by electrophoresis through 1.5% agarose gel stained with ethidium bromide and were photographed under UV light.

### The sensitivity, specificity, and reproducibility of the UNDP-PCR assay for TGEV

TGEV genomic RNA was extracted using RNA Kit and reverse transcribed to synthesize cDNA. Then, the part of TGEV ORF1a gene was amplified from cDNA using the primers (5’-CGTAATGGTGACAGCCGAT-3’/5’-AGCAGCATCACGGAAACCAT-3’). The 435 bp amplified products were cloned into pMD-19T simple Vector (TAKARA, Japan) and sequenced. The concentration of the plasmid was measured by Nanodrop 2000 Spectrophotometer (Thermo scientific, USA). The formula: amount (copies/μl) = 6.02×10^23^ (copies/mol) × concentration (g/μl)/MW (g/mol) was used to calculate the plasmid copy number. The viral copy number in samples per ml was tested by real-time PCR with serially diluted plasmid from 10^3^ to 10^9^ copies/ml. To test the sensitivity, the quantitative serum samples were diluted serially from 10 to 10^4^ copies/ml, and then detected by UNDP-PCR and conventional RT-PCR. The specificity of UNDP-PCR was tested through comparing with PCV2, PPV, PRRSV, PEDV and CSFV. Inter-assay and intra-assay were performed in three replicates by testing 3 different concentrations of diluted serum samples (5×10^3^ copies/ml, 5×10^2^ copies/ml, 50 copies/ml) for three consecutive days to test reproducibility of the UNDP-PCR assay for TGEV.

### The sensitivity of duplex UNDP-PCR assay for DNA and RNA viruses

To test the sensitivity of duplex UNDP-PCR assay for TGEV and PCV2, the serum samples containing TGEV or PCV2 were diluted serially from 10^4^ to 1 copies/ml respectively. The diluted samples containing same viral copy numbers of TGEV and PCV2 per ml were mixed, and then tested by single MMPs-based and duplex MMPs-based duplex UNDP-PCR, or conventional PCR/RT-PCR.

### The specificity and reproducibility of duplex UNDP-PCR assay for DNA and RNA viruses

In the study of evaluating the specificity of duplex UNDP-PCR, PPV, PEDV, PRRSV and CSFV were tested by the established method. The inter-assay and intra-assay tests were carried out in triplicate by detecting three different concentrations of mixed serum containing serial diluted TGEV and PCV2 (2000, 200, 20 copies/ml) to evaluate the reproducibility of this assay.

## Results

### Design and optimization of duplex UNDP-PCR assay

**Fig 1:**
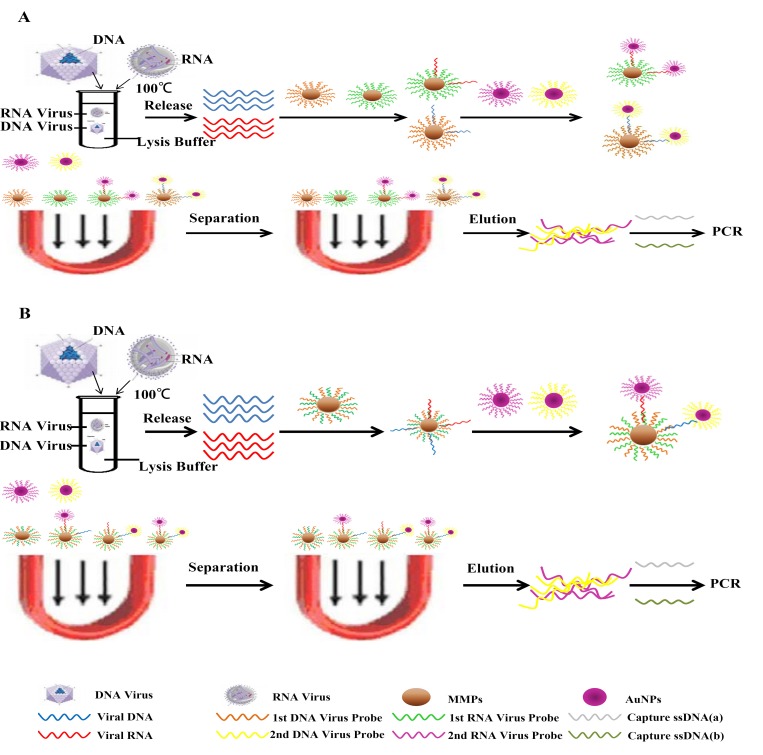
Schematic of the duplex UNDP-PCR assay. (A) Schematic of the single MMPs-based duplex UNDP-PCR assay. (B) Schematic of the duplex MMPs-based duplex UNDP-PCR assay.

To find a rapid and ultrasensitive diagnosis method for preclinical mixed infection of DNA and RNA viruses, a series of related experiments were performed to establish two kinds of protocol for duplex UNDP-PCR assay as schematically depicted in Fig 1.

In the previous study, we have optimized and established a UNDP-PCR method for DNA virus PCV2, which can detect 10 copies/ml of PCV2 serum sample and exhibited high specificity and reproducibility [19]. In the present study, PCV2 and TGEV are selected as representative DNA and RNA viruses, respectively. Therefore, a UNDP-PCR method for RNA virus TGEV needs to be established first.

**Fig 2:**
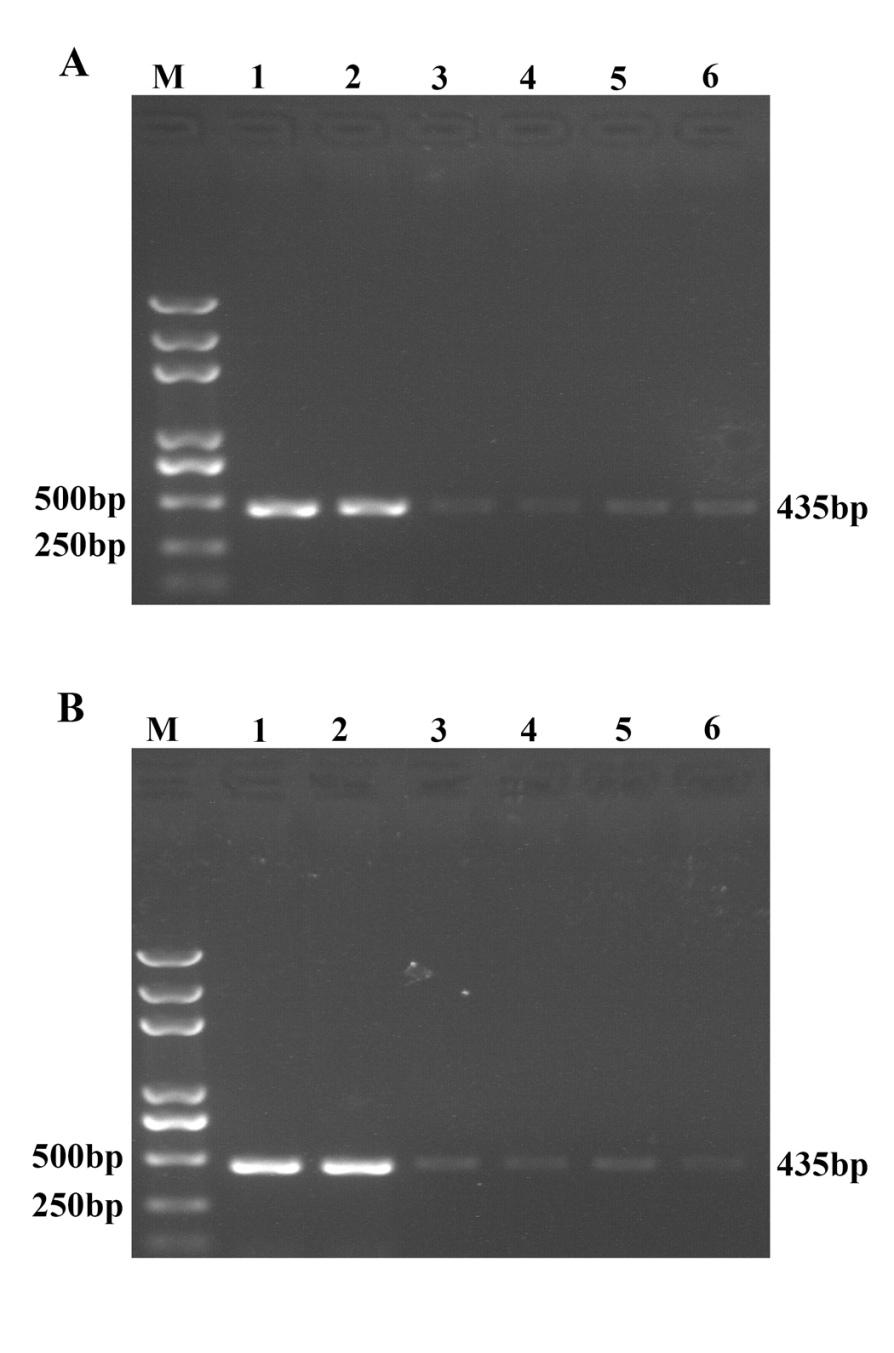
Comparison of different probes-functionalized MMPs and oligos-functionalized AuNPs. (A) Functional magnetic microparticles MMP-p1, -p2, -p3, -p4, -p5 and -p6 were incubated with RNA of TGEV in hybridization buffer at 40°C for 30 minutes, followed by washing and magnetic separation. Then the MMP-RNA complexes were reverse transcribed into cDNA, which were detected by TGEV-specific RT-PCR. M: Trans 2K Plus DNA Marker; 1: MMP1; 2: MMP2; 3: MMP3; 4: MMP4; 5: MMP5; 6: MMP6. (B) TGEV RNA was incubated with oligo1, oligo2, oligo3, oligo4, oligo5 or oligo6 functionalized Au-NPs at 50°C for 40 min. Then the complexes were washed and precipitated by centrifugation, followed by reverse transcription and TGEV specific RT-PCR detection. M: Trans 2K Plus DNA Marker; 1: oligo1; 2: oligo2; 3: oligo3; 4: oligo4; 5: oligo5; 6: oligo6.

TGEV, an enveloped virus of the *coronaviridae* family, contains a single-stranded positive-sense RNA genome of 28.5 kb. The first two-thirds of the viral genome encodes two replicases and only exists in genomic RNA, while the last third exists in both genomic RNAs (gRNA) and subgenomic mRNAs (sgmRNAs) to encode structural and nonstructural proteins of virus [21, 29, 30]. In this assay, we first quantified viral number using primers targeted to TGEV gRNA and different sgmRNAs and found that viral numbers were significantly different when the primers were targeted to TGEV gRNA and different sgmRNAs (data not shown). Therefore, we designed probes targeted to replicase protein-encoding region ORF1a to quantify the amount of TGEV gRNA. Six targeted regions were selected from the 5’ end, middle and 3’ end of the ORF1a for designing probes 1 to 6 (Table 1). All the targeted sequences of these probes are highly conserved in variety of TGEV strains. Probes 1 to 6 were coated to magnetic microparticles to prepare functional magnetic beads MMP-p1, -p2, -p3, -p4, -p5 and -p6, respectively. To select the optimal probes for capturing of TGEV genomic RNA, capture efficiency of these designed probes were determined by conventional RT-PCR assay. The results showed that all six probes could capture TGEV RNA, however MMP-p1 and MMP-p2 exhibited higher capture ability for TGEV RNA (Fig 2A). Next, we assessed capture efficiency of functionalized gold nanoparticles coated with oligonucleotides (oligo) 1 to 6 that shared same targeted sequence with probe 1 to 6. The prepared functionalized Au-NPs were precipitated by centrifugation and were detected by RT-PCR assay. The results showed that oligo 1 and 2-coated Au-NP possessed higher binding ability with TGEV nucleic acid (Fig 2B). Therefore, in the UNDP-PCR assay for TGEV, probe 1-functionalized MMPs and oligo 2-functionalized Au-NPs are optimal for capture of TGEV RNA and formation of sandwich complex.

### The sensitivity of UNDP-PCR for RNA viruses (TGEV)

**Fig 3:**
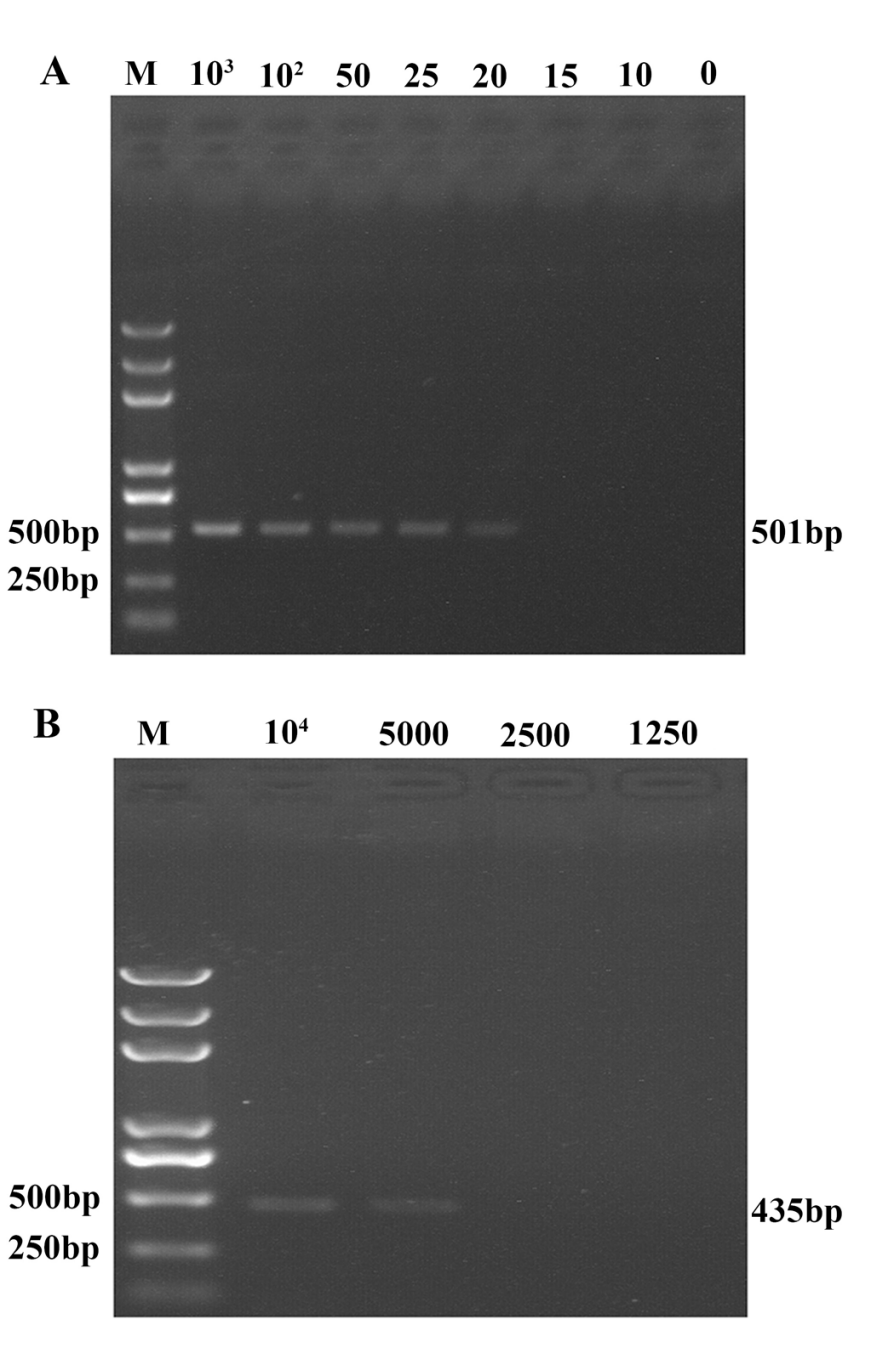
Analysis of the sensitivity of UNDP-PCR for TGEV. (A) Serial dilutions of TGEV serum samples were detected by UNDP-PCR for TGEV. (B) Serial dilutions of TGEV serum samples were detected by conventional RT-PCR.

Serial 10-fold dilutions of TGEV in serum samples were tested to assess the sensitivity of UNDP-PCR for RNA virus. TGEV genomic RNA was released by boiling with lysis buffer with RNase inhibitors and was used to form AuNP-RNA-MMP complexes, followed by magnetic separation and oligonucleotide elution. The oligonucleotides were then purified and detected by UNDP-PCR. As shown in Fig 3A, visible targeted bands around 501 bp could be seen in lanes representing serum samples with viral concentrations ranging from 10^3^ copies/ml to 20 copies/ml respectively, but it could not be detected in the negative control serum without TGEV and the TGEV serum sample below 20 copies/ml, indicating that the detection limit of UNDP-PCR assay for TGEV was 20 copies/ml in serum sample. However, at least 5000 copies/ml of TGEV serum sample was able to be detected by conventional RT-PCR assay (Fig 3B), suggesting that the sensitivity of UNDP-PCR specific for TGEV was 250-fold that of the conventional RT-PCR for TGEV.

### Reproducibility and specificity of UNDP-PCR for RNA viruses (TGEV)

**Fig 4:**
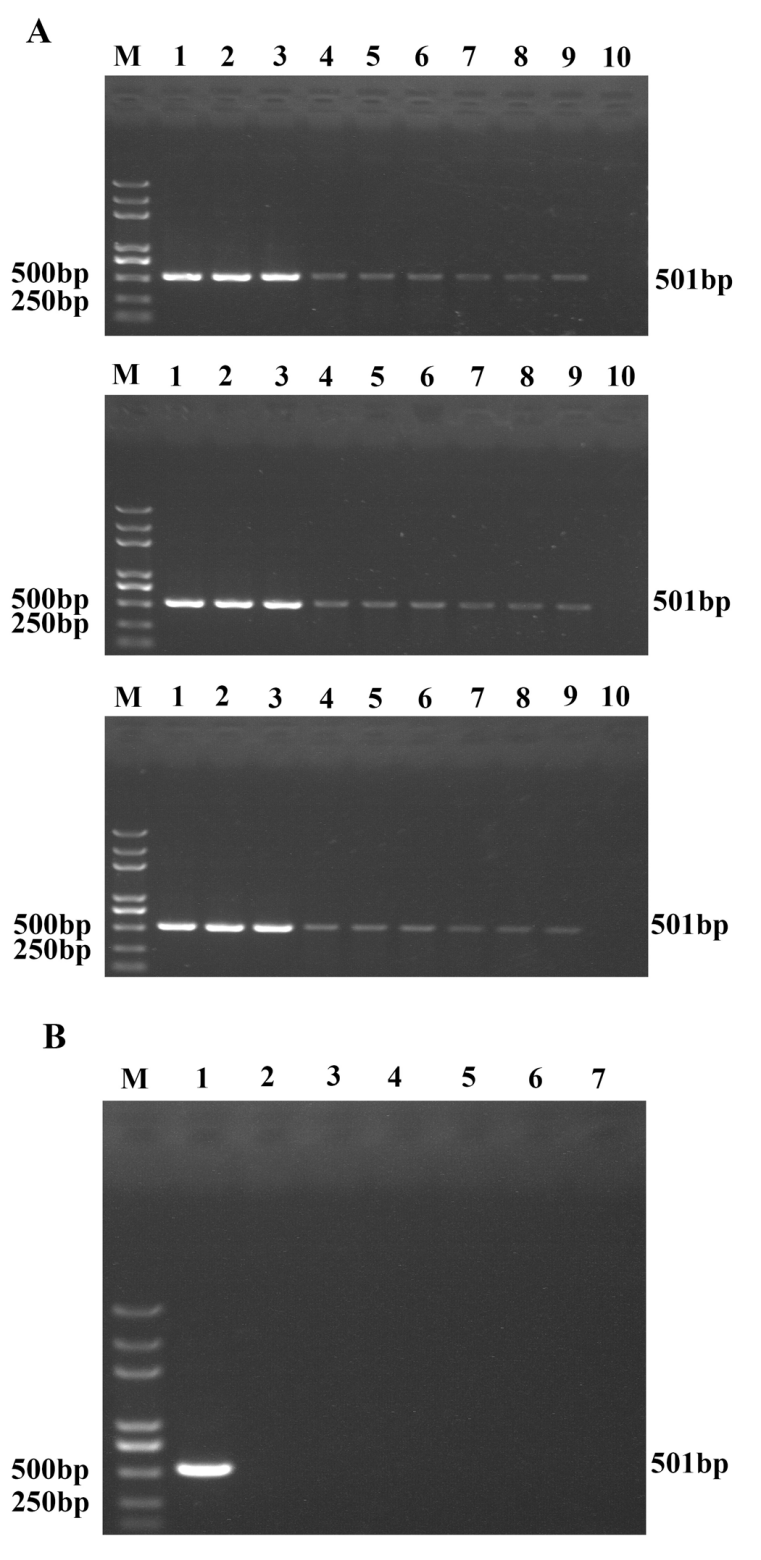
Analysis of the reproducibility and specificity of UNDP-PCR for TGEV. (A) Three different concentrations of TGEV serum samples were detected by UNDP-PCR for TGEV in triplicates and in three independent runs. Lane M: Trans 2K Plus DNA Marker; lane 1–3: 5×10^3^; lane 4–6: 5×10^2^; lane 7–9: 5×10^1^; lane10: negative samples. (B) PCV2, PPV, PEDV, CSFV, PRRSV and blood collected from healthy swine were detected by UNDP-PCR for TGEV as control. Lane M: Trans 2K Plus DNA Marker; lane 1: TGEV; lane 2: the blood of healthy swine; lane 3: PCV2; lane 4: PPV; lane 5: PEDV; lane 6: PRRSV; lane 7: CSFV.

The reproducibility of UNDP-PCR for TGEV was estimated by three independent runs for three consecutive days, with triplicates of each concentration (5×10^3^ copies/ml, 5×10^2^ copies/ml, 50 copies/ml). Consistent results of UNDP-PCR were obtained in the inter-assay and intra-assay test (Fig 4A).

PPV, PCV2, PRRSV, CSFV, PEDV, TGEV and the serum collected from healthy pigs were used to evaluate the specificity of the established UNDP-PCR for TGEV. In the Fig 4B, the UNDP-PCR detection only appeared to be positive with TGEV, whereas no specific PCR products of 501 bp were obtained from the assay using PCV2, CSFV, PRRSV, PEDV, PPV or healthy pigs serum as pending samples, which indicated that UNDP-PCR assay had high specificity for TGEV and did not show cross-reactivity with PCV2, CSFV, PRRSV, PEDV and PPV.

### The sensitivity of duplex UNDP-PCR for both DNA and RNA viruses

**Fig 5:**
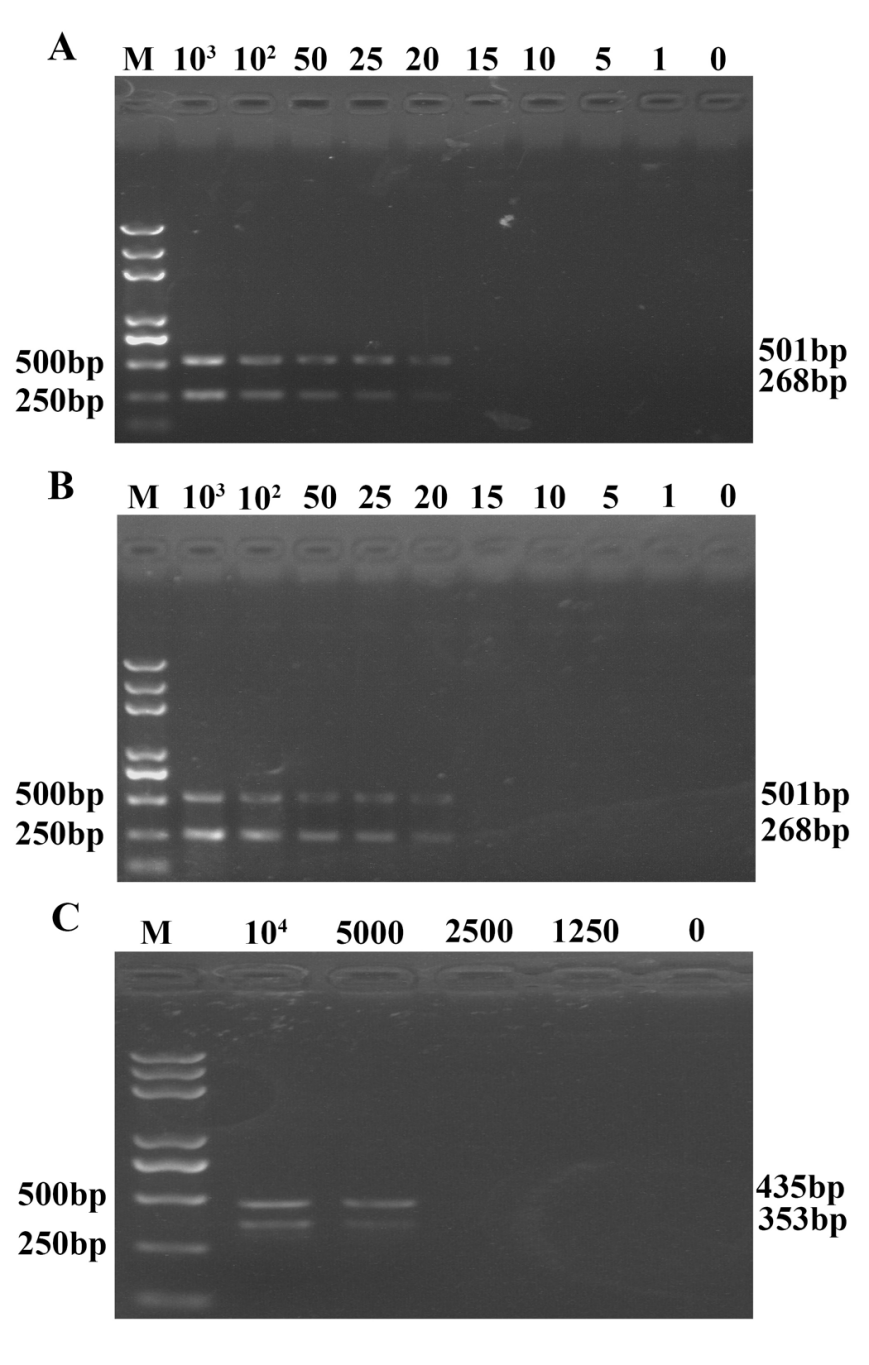
Sensitivity of single MMPs-based and duplex MMPs-based duplex UNDP-PCR assay for simultaneous detection of DNA and RNA viruses. (A) The serum samples of PCV2 and TGEV were diluted serially and mixed as templates for single MMP-based duplex UNDP-PCR assay. (B) Mixed quantified serum samples of PCV2 and TGEV were tested by duplex MMPs-based duplex UNDP-PCR assay. (C) The serum samples of PCV2 and TGEV were diluted serially, and the DNA of PCV2 and RNA of TGEV were extracted as the template for conventional duplex PCR/RT-PCR detection.

To compare the sensitivity of single MMPs-based and duplex MMPs-based duplex UNDP-PCR assay for simultaneous detection of TGEV and PCV2 in same reaction assay system, qualified serum samples of TGEV and PCV2 were diluted serially with the range from 1 to 10^3^ copies/ml. Then the diluted samples containing TGEV and PCV2 were mixed as the template for testing the sensitivity of single MMPs-based and duplex MMPs-based duplex UNDP-PCR assay. As shown in Fig 5A and 5B, the detection limits of single MMPs-based and duplex MMPs-based duplex UNDP-PCR were identical with 20 copies/ml for TGEV and PCV2 in serum. However, at least 5000 copies/ml of PCV2 and TGEV serum sample was able to be detected by conventional duplex PCR/RT-PCR assay (Fig 5C). These results suggested that the sensitivity of duplex UNDP-PCR specific for PCV2 and TGEV was 250-fold that of the conventional duplex PCR/RT-PCR for these two viruses, and that MMPs coated with one virus probe or two virus probes did not affect the capture efficiency of functionalized MMPs and the sensitivity of UNDP-PCR assay.

### The specificity and reproducibility of duplex UNDP-PCR for both DNA and RNA viruses

**Fig 6:**
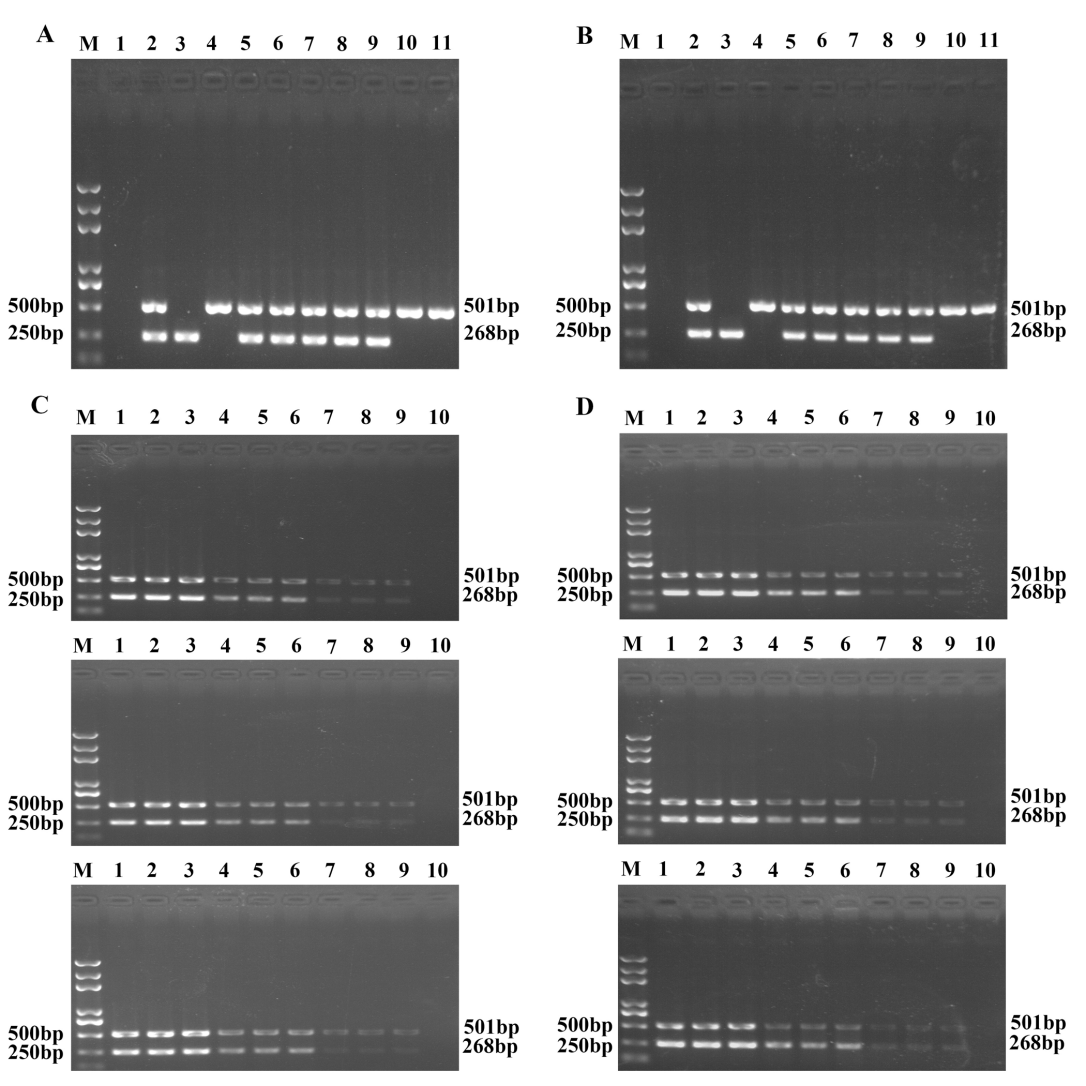
Specificity and reproducibility of the single MMPs-based and duplex MMPs-based duplex UNDP-PCR assay. Two viruses (TGEV and PCV2) as well as unrelated viruses (PPV, CSFV, PEDV, PRRSV), and serum of healthy pigs were tested to evaluate the specificity of the single MMPs-based and duplex MMPs-based duplex UNDP-PCR. Three different concentrations of mixed serum containing serial diluted TGEV and PCV2 were used to evaluate the reproducibility of the single MMPs-based and duplex MMPs-based duplex UNDP-PCR assay in triplicates and in three independent runs. (A) Agarose gel electrophoresis of the single MMPs-based duplex UNDP-PCR products. (B) Agarose gel electrophoresis of the duplex MMPs-based duplex UNDP-PCR products. Lane M: Trans 2K Plus DNA Marker; lane 1: blood of healthy swine; lane 2: PCV2, TGEV, PPV, CSFV, PEDV and PRRSV; lane 3: PCV2; lane 4: TGEV; lane 5: PCV2 and TGEV; lane 6: PCV2, TGEV and PPV; lane 7: PCV2, TGEV and CSFV; lane 8: PCV2,TGEV and PEDV; lane 9: PCV2,TGEV and PRRSV; lane 10: TGEV and PPV; lane 11: TGEV and PEDV. (C) Analysis of the reproducibility of the single MMPs-based duplex UNDP-PCR. Lane M: Trans 2K Plus DNA Marker; lane 1–3: 2×10^3^; lane 4–6: 2×10^2^; lane 7–9: 2×10^1^; lane 10: negative samples. (D) Analysis of the reproducibility of the duplex MMPs-based duplex UNDP-PCR. Lane M: Trans 2K Plus DNA Marker; lane 1–3: 2×10^3^; lane 4–6: 2×10^2^; lane 7–9: 2×10^1^; lane 10: negative samples.

To evaluate the specificity of single MMPs-based and duplex MMPs-based duplex UNDP-PCR for both DNA and RNA viruses, magnetic beads coated with specific probes for TGEV and PCV2 alone or together and gold nanoparticles coated with specific oligos for TGEV and PCV2 alone were added to one single reaction tube. As shown in Fig 6A and 6B, both assays were specific to the target viruses, producing specific amplified products (501 bp for TGEV and 268 bp for PCV2). No amplicons were yielded with PPV, PRRSV, CSFV, PEDV and samples from health pigs. Three independent replicates assay of both assays gave highly consistent results respectively (Fig 6C and 6D).

### Application of duplex UNDP-PCR for detecting virus infection in field

Pre-clinical serum samples from epidemic farms without diseased pigs were detected for TGEV and PCV2 using duplex UNDP-PCR assay and conventional duplex PCR/RT-PCR. Conventional duplex PCR/RT-PCR detection showed that among 162 samples, 7 samples were PCV2 positive, 2 samples were TGEV positive, but none of samples were found to be positive for both PCV2 and TGEV. Whereas duplex UNDP-PCR assay showed that 48 samples were PCV2 positive, 15 samples were TGEV positive, 6 samples were positive for both PCV2 and TGEV (Table 2). The positive samples detected by the duplex UNDP-PCR method included all of the samples found to be positive by the conventional duplex PCR/RT-PCR. The positive detection rate of duplex UNDP-PCR was 29.6% for PCV2, 9.3% for TGEV and 3.7% for PCV2 and TGEV mixed infection, which increased 29% and 11.7% for PCV2 and TGEV preclinical infection than that of conventional duplex PCR/RT-PCR (*p* < 0.01). As shown in Table 2, the results were consistent when single MMPs-based and duplex MMPs-based duplex UNDP-PCR were used to detect these samples, suggesting two kinds of duplex UNDP-PCR possessed same detection efficiency.

**Table 2:** The detection rate of PCV2 and/or TGEV infected preclinical samples using conventional PCR and RT-PCR, single MMPs-based UNDP-PCR and duplex MMPs-based UNDP-PCR methods.

	Duplex UNDP-PCR		Conventional duplex PCR/ RT-PCR
	single MMPs-based	duplex MMPs-based	
Total number of tested samples	162	162	162
PCV2 positive samples	48	48	7
TGEV positive samples	15	15	2
PCV2+TGEV positive samples	6	6	0
PCV2 positive rate (%)	29.6	29.6	4.3
TGEV positive rate (%)	9.3	9.3	1.2
PCV2+TGEV positive rate (%)	3.7	3.7	0

**Fig 7:**
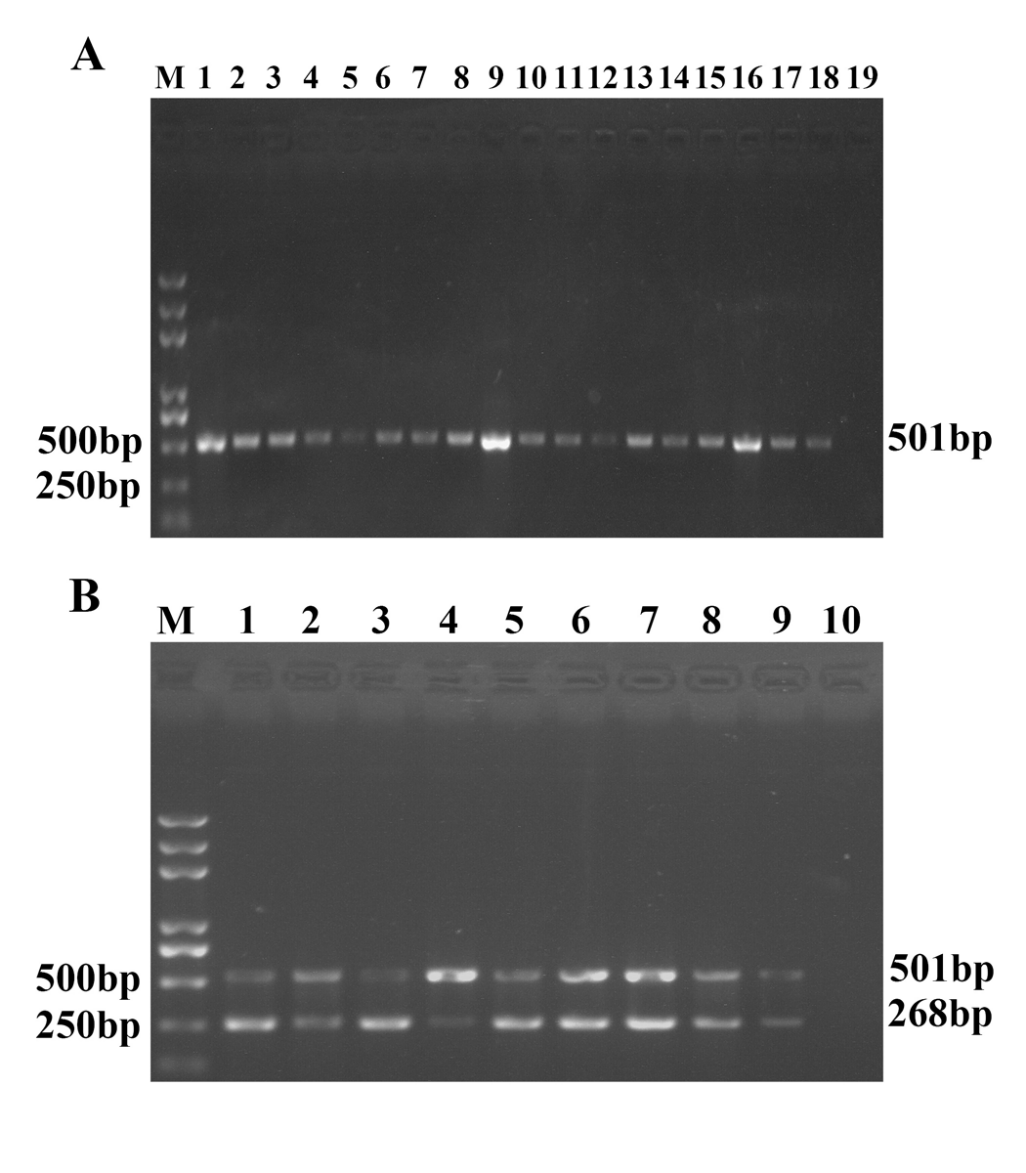
Detection of preclinical samples using duplex UNDP-PCR for TGEV and PCV2. (A) Agarose gel electrophoresis analysis of the relative viral load levels of 15 TGEV positive preclinical specimens identified by duplex UNDP-PCR. Lane M: Trans 2K Plus DNA Marker; lane 1–15: preclinical specimens; lane 16: 5×10^3^ Standards; lane 17: 5×10^2^ Standards; lane 18: 5×10^1^ Standards; lane 19: negative samples. (B) Agarose gel electrophoresis analysis of the relative viral load levels of 6 PCV2 and TGEV double positive preclinical samples identified by duplex UNDP-PCR. Lane M: Trans 2K Plus DNA Marker; lane 1–6: preclinical specimens; lane 7: 5×10^3^ Standards; lane 8: 5×10^2^ Standards; lane 9: 5×10^1^ Standards; lane 10: negative samples.

Next, the relative band intensities of the unknown samples were compared with that of standard virus samples (50, 500 and 5000 copies/ml PCV2 and TGEV) to evaluate the viral loads of PCV2 and TGEV in all of positive samples. Among 48 PCV2 positive samples, 7 samples were over 5000 copies/ml, 15 samples were between 5000 and 500 copies/ml, 23 samples were between 500 and 50 copies/ml, and 3 samples were below 50 copies/ml in viral load (Data not shown); among 15 TGEV positive samples, 2 samples were over 5000 copies/ml, 5 samples were between 5000 and 500 copies/ml, 6 samples were between 500 and 50 copies/ml, and 2 samples were below 50 copies/ml (Fig 7A). Among 6 PCV2 and TGEV double positive samples, the PCV2 viral loads of 4 samples were between 5000 and 500 copies/ml, 1 sample was between 500 and 50 copies/ml, and 1 sample was below 50 copies/ml, the TGEV viral loads of 3 samples were between 5000 and 500 copies/ml, 2 sample were between 500 and 50 copies/ml, and 1 sample was below 50 copies/ml (Fig 7B).

Taken together, this UNDP-PCR assay was found to be the best method for detecting DNA and RNA viruses in preclinical samples simultaneously. All the results indicated that UNDP-PCR-based assay was a rapid and economical approach with high specificity and high sensitivity.

## Discussion

To date, traditional approaches to detect mixed infection of RNA and DNA viruses present some limitations, such as complex virus genome isolation procedures, limited sensitivity, narrow detection range and lack of specific antibodies for different viruses, which lacks the effective monitoring of swine infectious diseases. Although uniplex and multiplex PCR assays were developed and tried to detect RNA and DNA virus mixed infection simultaneously, in fact, to extract viral RNA and DNA and reverse transcription of RNA into cDNA are still time-consuming and labor intensive [9, 31]. In addition, low virus titer in early stage of infection is not able to be detected by conventional PCR or RT-PCR, failing in timely diagnosis of infection. Viruses are more likely to spread across piggeries and cause more sickness and death in piglets, which will bring huge threat to pig industry [32]. With the development of nanotechnology, the UNDP-PCR based on nanoparticle and DNA probe makes it possible to detect DNA and RNA viruses simultaneously especially in subclinical infection without the need for nucleic acid extraction separately [33].

Duplex UNDP-PCR assay gains more merits over established traditional approaches for RNA/DNA virus diagnosis. The first advantage that should be mentioned is that duplex UNDP-PCR is more time-saving and cost-effective than other routine PCR-based methods. In this study, field samples collected from pig-producing farms were boiled with lysis buffer for 15 min to release viral genome, so there is no need to extract nucleic acid using DNA/RNA extraction kits. Particularly, unlike other PCR-based assays, extracted and purified RNA need to be reverse transcribed into cDNA. Hence, the whole detection process could be completed in short period of time. Secondly, this assay could detect DNA and/or RNA virus in serum samples with an extreme low concentration in preclinical infection. Accordingly, functionalized magnetic beads and gold nanoparticles which were coated with TGEV and/or PCV2 specific probes and oligonucleotides targeting two distinct virus genomic sequences were added to form a sandwich complex. In each binding events, the gold nanoparticles carried with large number of oligonucleotides, which could be used as templates for subsequent PCR assay. Thus, weak signals from extreme low concentration of samples were highly amplified. In addition, the duplex UNDP-PCR could detect DNA and RNA virus from large-scale serum samples simultaneously. In the reaction system, magnetic beads and gold nanoparticles coated with single and/or duplex specific probes for TGEV and PCV2 were added to capture and enrich viral nucleic acid, so this approach could detect infection of TGEV and PCV2 alone or together in a single reaction tube.

The results showed that duplex UNDP-PCR (single MMPs-based and duplex MMPs-based) developed in the study was able to detect 20 copies for PCV2 and TGEV. The sensitivity of duplex UNDP-PCR was approximately 250-fold that of conventional duplex PCR/RT-PCR. In terms of specificity, specific probes and oligonucleotides for TGEV and PCV2 were assessed through testing capture efficiency and specificity of different probes- or oligonucleotides-coated magnetic beads or gold nanoparticles. As a consequence, duplex UNDP-PCR showed high specificity with TGEV and PCV2, yielding different size of PCR products (501 bp and 268 bp respectively). The results of detection for preclinical samples indicated that duplex UNDP-PCR assay (29.6% for PCV2, 9.3% for TGEV and 3.7% for PCV2 and TGEV mixed infection) described here was more sensitive than conventional detection methods (4.3% for PCV2, 1.2% for TGEV and 0% for PCV2 and TGEV mixed infection).

## Conclusion

The duplex UNDP-PCR assay developed in this study provided a useful tool for simultaneous detection of RNA (TGEV) and DNA viruses (PCV2) without the need for viral nucleic acid extraction, purification and reverse transcription. This assay could increase positive detection rates of virus infection and is useful to evaluate the viral loads in pre-clinically infected samples. In summary, the duplex UNDP-PCR assay is an economical and rapid detection approach with high specificity and sensitivity.
